# Assessing optimal: inequalities in codon optimization algorithms

**DOI:** 10.1186/s12915-021-00968-8

**Published:** 2021-02-19

**Authors:** Matthew J. Ranaghan, Jeffrey J. Li, Dylan M. Laprise, Colin W. Garvie

**Affiliations:** Center for the Development of Therapeutics, The Broad Institute of MIT and Harvard, 415 Main Street, Cambridge, MA 02142 USA

**Keywords:** Codon optimization, Codon usage, Algorithm, Violin plot, %MinMax, KRas4B

## Abstract

**Background:**

Custom genes have become a common resource in recombinant biology over the last 20 years due to the plummeting cost of DNA synthesis. These genes are often “optimized” to non-native sequences for overexpression in a non-native host by substituting synonymous codons within the coding DNA sequence (CDS). A handful of studies have compared native and optimized CDSs, reporting different levels of soluble product due to the accumulation of misfolded aggregates, variable activity of enzymes, and (at least one report of) a change in substrate specificity. No study, to the best of our knowledge, has performed a practical comparison of CDSs generated from different codon optimization algorithms or reported the corresponding protein yields.

**Results:**

In our efforts to understand what factors constitute an optimized CDS, we identified that there is little consensus among codon-optimization algorithms, a roughly equivalent chance that an algorithm-optimized CDS will increase or diminish recombinant yields as compared to the native DNA, a near ubiquitous use of a codon database that was last updated in 2007, and a high variability of output CDSs by some algorithms. We present a case study, using KRas4B, to demonstrate that a median codon frequency may be a better predictor of soluble yields than the more commonly utilized CAI metric.

**Conclusions:**

We present a method for visualizing, analyzing, and comparing algorithm-optimized DNA sequences for recombinant protein expression. We encourage researchers to consider if DNA optimization is right for their experiments, and work towards improving the reproducibility of published recombinant work by publishing non-native CDSs.

**Supplementary Information:**

The online version contains supplementary material available at 10.1186/s12915-021-00968-8.

## Background

**Fig. 1:**
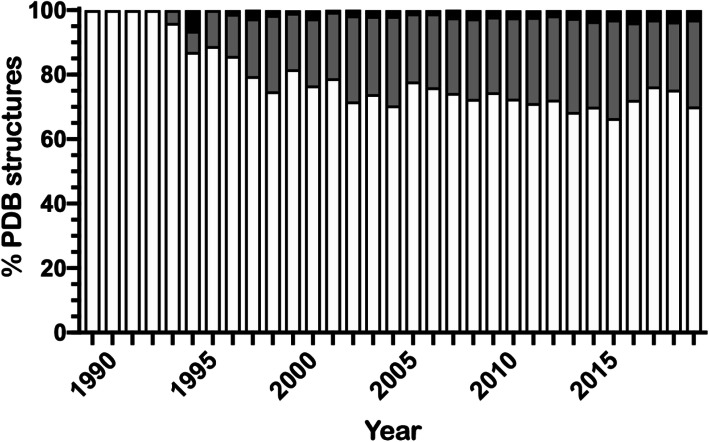
Normalized histogram of structures from the RCSB Protein Databank (PDB) for recombinant proteins from *Homo sapiens* by mammalian (black), insect (gray), and *E. coli* (white) expression systems

Structure-based drug design and high-throughput compound screening typically requires hundreds of milligrams of soluble, high-purity protein. A recent analysis of three structure-based consortia highlighted the challenges in generating sufficient protein to enable structural studies, with an estimated > 70% failure rate of cloned targets resulting in a purified product and only 3% success rate in leading to the submission of PDB structures [1]. Many protein targets are generated through heterologous expression of recombinant proteins. Bacterial hosts, specifically *Escherichia coli*, are the most commonly used expression system over eukaryotic systems because they are more economical, have easily manipulated genetics, and offer faster growth rates to achieve high cellular densities. The popularity of *E. coli* is reflected in the RCSB (Research Collaboratory for Structural Bioinformatics), with 73 ± 3% of human proteins being made in *E. coli* since 2000 (Fig. 1), and in the development of several new commercial expression strains over the last decade [2].

The biological principles for recombinant protein expression are well established; however, the ability to distinguish protein targets that express well from those that express poorly is still considered a “black box” process that often requires screening many conditions to obtain a soluble product. Among the numerous components that contribute to recombinant expression, there is empirical evidence that manipulation of the following variables can improve yields of soluble protein product: the bacterial strain [2]; expression media [3, 4]; genetic fusion of the target protein to a purification tag, carrier protein, or chaperone [5, 6]; and altering of the coding DNA sequence (CDS) [5, 7]. Reconfiguring the CDS from the native sequence to one optimized for expression in a particular host system has become almost commonplace over the last 20 years. Most commercial vendors offer a custom optimization algorithm and several online servers exist (Table 1 in reference [1]).

**Table 1:** Descriptions of codon usage databases for either the generic class or B strain of *E. coli*. Each annotation describes the source of the genetic data, the total number of coding DNA sequences (CDS) extracted from the gene source(s), and the number of codons extracted from genes used to construct each database

Author or database	***E. coli*** strain	Gene source	# CDS	# codons
Sharp and Li [8] ^a^	Generic	GenBank	27	6240
			15	9223
			57	25,010
			58	22,612
Kazusa database [9]	Generic	GenBank	8087	2,330,943
	B	GenBank	11	3771
HIVE-CUT database [10]	Generic	GenBank and RefSeq	68,262,063	20,219,118,236
	B	GenBank and RefSeq	13,042	3,953,593
GtRNAdb [11, 12]	Generic	GenBank and RefSeq	5011	1,538,003
GenScript^b^	Proprietary	Undefined	Undefined	Undefined
Dong et al. [13]	W1485 (K12)	N/A	Total RNA	Undefined

^a^Authors divided their dataset into four groups that represent genes that exhibit “very high expression,” “high levels of expression,” “moderate codon bias,” or “low codon bias” that are represented from the top down, respectively

^b^www.genscript.com

Despite the general acceptance of working with synthetic CDSs, there is little guidance for what defines an optimized sequence. Differently optimized DNAs are reported to influence recombinant protein activity [14], which aligns well with observations of aberrant effects from “silent” mutations that change substrate specificity or induce disease [15]. Some empirical rules and guidelines for optimizing CDSs have been published [16, 17], but appear to be anecdotal and most impactful in unicellular expression systems [18]. For the purpose of this work, we define an optimized CDS as one that produces the maximal expression of soluble protein in order to enable investigation of the biological function of the molecule. The goal of the work presented here is to identify inconsistencies between several commonly used and publicly available codon optimization algorithms, to outline parameters that researchers should consider when enhancing CDSs for recombinant protein expression, and to encourage researchers to publish the DNAs used in their work as supporting information, in publicly available databases (e.g., DNASU, SGDB) [19, 20], or in a vector repository (e.g., Addgene). None of these efforts are common practice and such inconsistencies could minimize the challenges often encountered when attempting to reproduce published results. We also stress that we make no claims of the quality of DNA products from commercial vendors and expect commercial and public optimization algorithms to evolve over time.

## Results

### Analysis of codon bias and published codon usage tables

**Fig. 2:**
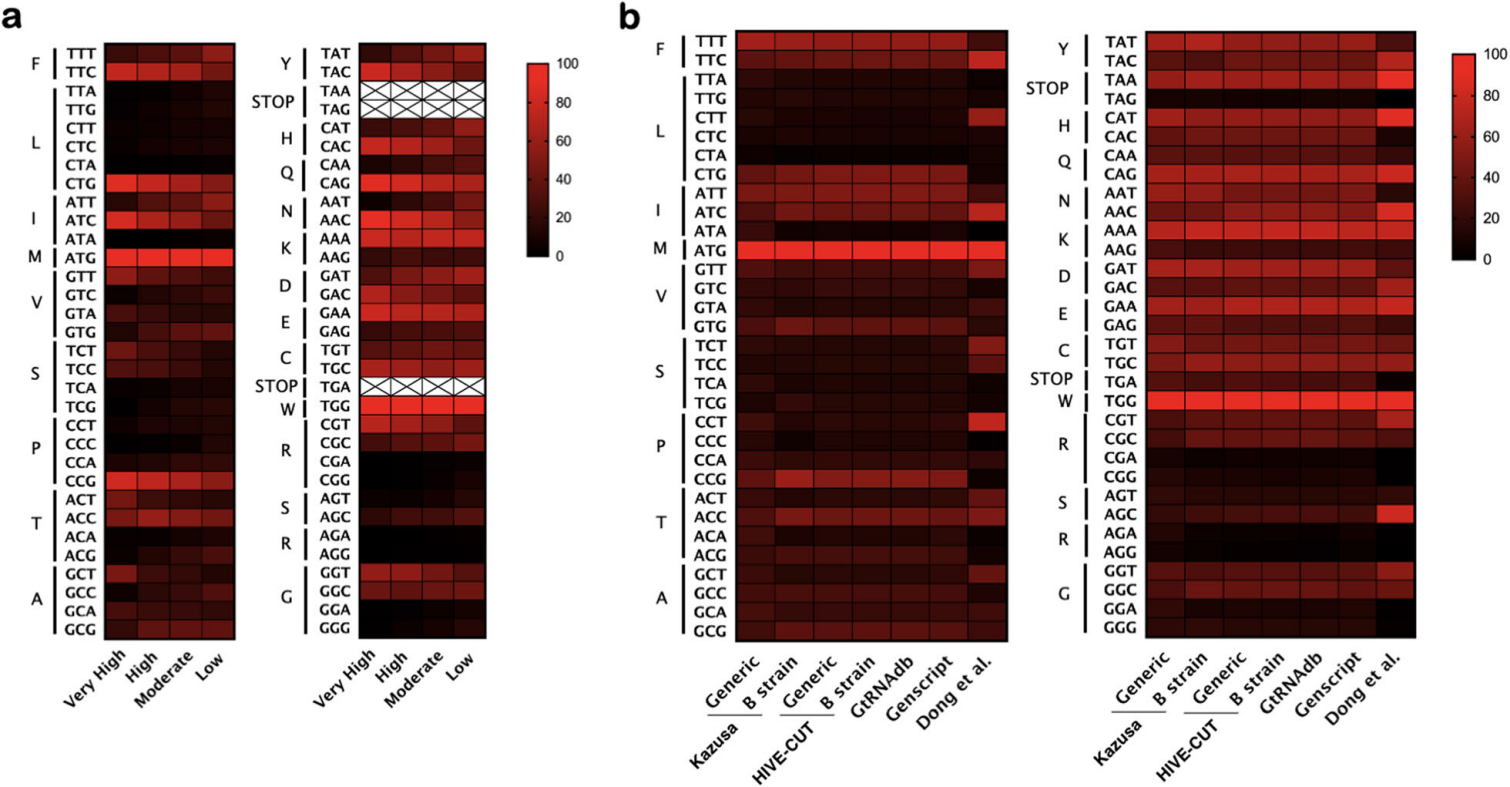
Heat map of the relative codon frequencies for *E. coli* grouped by amino acid. **a** Values from Sharp and Li (1986). The authors binned their datasets (see Table 1) into four groups as shown in the figure. An “X” represents no available data for the codon. **b** Codon distributions for various codon usage databases or datasets described in Table 1. Data for Dong et al. are from the growth rate at 2.5 h^−1^

Synthetic DNA sequences enable biomedical researchers to manipulate genetic sequences on a scale from a single nucleotide to megabase genomes [21, 22]. The cost of synthetic DNA has dramatically decreased over the last several decades and often replaces traditional cloning methods, like PCR amplification of native cDNA, for heterologous expression of recombinant proteins. This shift has led to the rise of artificial sequences that use codon degeneracy to alter the DNA with synonymous codons that are more frequently used in the expression host. Known as codon bias [16], this approach stems from the observation that genes with high expression levels typically contain few rare codons, whereas low expressing genes have a broader distribution of rare and frequent codons. Sharp and Li originally proposed a system where synonymous codons were normalized for each amino acid and used to calculate the relative synonymous codon usage (RCSU) based on their codon usage to one another within a gene [8]. This is a normalized scale of 0 (codon never used) up to 100 (codon used 100% of the time) that represents the distribution of synonymous codons for a particular amino acid. They then classified the 157 available genes (65,547 codons) from Genbank into four classes (Table 1): 27 “very highly expressed” genes, 15 “highly expressed” genes, 57 genes with “moderate codon bias”, and 58 genes with “low codon bias”. We present the codon-specific data as a heat map, rather than table format, for visualization of the tRNA codon relative to each amino acid (Fig. 2a). Note that two amino acids, methionine (M; ATG) and tryptophan (W; TGG), have only one tRNA codon and retain a value of 100 across the heat map. All other amino acids have multiple codons representing them. For example, there are three codons for isoleucine (I) with one codon (ATA) being underrepresented in all classes (< 5%) and two codons (ATT and ATC) steadily transition from well separated in highly expressing proteins (16% and 84%, respectively) to roughly equivalent usages in lower expressing proteins (55% and 41%, respectively). Many amino acids share similar trends, with one codon dominating the others in highly expressing proteins, but explanations for this phenomenon remain speculative.

This RCSU scale led to the development of the Codon Adaptation Index (CAI) metric that serves as a measure of the codon bias of a CDS relative to highly expressed genes (e.g., ribosomal proteins, transcription factors) [23]. Mathematically, CAI represents the geometric mean of the RCSU value for each codon within the gene relative to that of the most commonly used synonymous codon (highest possible RCSU value). Other algorithms have been developed over time [24, 25] and their merits are discussed elsewhere [26], but CAI remains the most widely used metric for representing codon bias. Despite several reports showing no correlation between the CAI and protein expression levels [27, 28], many commercial synthetic DNA vendors still openly employ this method for calculating codon bias. We note that some modern algorithms appear to generalize codon usage without any clear definition of genes used to assign bias by expression levels, which was the original basis of CAI defined by Sharp and Li [26]. Codon bias, however, is considered only one facet of these complex algorithms that include any combination of an ever-growing list of variables that include, but are not limited to minimizing restriction enzyme sites, %GC content, repeating sequences, codon-pair bias [29], 5′ mRNA structure or stability [30, 31], and internal ribosome binding sites [32]. As we will show in this work, the optimization algorithms from academic and commercial entities produce drastically different DNAs by using varying levels of these and occasionally other parameters, yet the common considerations are based on tRNA frequency tables.

The original codon usage tables proposed by Sharp and Li were based on a small number (< 200) of *E. coli* genes that were available through the GenBank database at the time [8]. Modern day access to a wide array of genomic data, cataloged in GenBank and RefSeq, has expanded the scope of codon usage to many millions of CDSs for organism-specific codon usage tables. The Kazusa database is the most commonly cited source for organism-specific codon frequency data in the literature [16, 33–36] and by commercial DNA vendors [37–39]. This database compiled the CDS from GenBank and was one of the more comprehensive databases for codon usage when created in 2000; however, it has not been updated since 2007 [9]. We therefore asked whether there were more updated databases for comparison with the Kazusa dataset and focused on *E. coli*, because of the widespread application to recombinant protein work (Fig. 1). A handful of databases were found in the literature (Table 1). The GtRNAdb database provides comprehensive, organism-specific codon usage tables, but the primary focus is providing a web-based resource for the non-coding tRNA genes [11, 12]. Additionally, proprietary codon usage tables exist from several commercial sources (e.g., GenScript), but are difficult to tabulate in a meaningful way as the underlying data are restricted. We determined that the HIVE-CUT database was the most comprehensive resource, with > 8000-fold more CDS than the Kazusa database, and is regularly updated via GenBank and RefSeq [10]. Visualization of these *E. coli* codon usage tables shows that the Kazusa database for generic *E. coli* frequencies has a more muted tone than other datasets when comparing amino acids with more than two codons (Fig. 2b). This equivalence within the Kazusa dataset signifies underrepresentation of the codon bias that is observed by most other datasets. For example, CCG (proline) and ACC (threonine) are the most highly represented codons within their respective sets for all but the Kazusa and Dong databases. The table from Dong et al. represents an outlier dataset because the RCSU values represent the total extracted cellular RNA in *E. coli* instead of only CDSs [13]. Moreover, there is a direct contrast between the Kazusa datasets for the generic and the underrepresented B strain of *E. coli*. The B strain is the most commonly used bacterial strain for recombinant protein expression.

Based on this analysis, we propose that researchers and commercial vendors use the more updated organism-specific codon usage tables from databases like HIVE-CUT instead of Kazusa. This switch is especially pertinent if one’s motivation for using the Kazusa database is using a large genetic database for a comprehensive representation of organism-specific genes. We will use the HIVE-CUT tables for our analysis in this work.

### Significant variability observed between optimization algorithms

The concept of codon optimizing a CDS for a particular expression system is generally understood to influence protein expression levels. This task is traditionally accomplished by substituting rare codons with more frequently used synonymous codons to maximize CAI values (see above). Such changes theoretically enable faster translation rates and higher recombinant protein levels while minimizing the chance of depleting tRNA pools that can lead to cellular stress. Various reports, summarized in Table 2, demonstrate that differently optimized CDSs can increase expression levels 2-fold over the native sequence for ~ 25% of evaluated targets [40, 41]. These studies can be classified into categories where the authors compare the following: native and optimized CDSs of up to 100 different genes [40, 41, 44], randomly substituting synonymous codons in a single gene [27], weighting various coding factors (e.g., translational speed, mRNA structure) [42, 43], or methodically testing up to 20 different optimized DNAs [28]. Most of these studies, however, report *optimization* routines that use CAI through various commercial vendors. More recent optimization strategies suggest that preserving evolutionarily conserved clusters of rare codons could influence protein folding or other maturation events (e.g., post-translational modification, chaperone recruitment) in the native host [45, 46]. “Codon harmonization,” in particular, attempts to match the codon frequency distribution throughout the native CDS with that of the expression host [47]. As we will show below, harmonization does not appear to be a driving force in present-day optimization algorithms, but remains an interesting concept that should be explored further.

**Table 2:** Summary of literature references comparing protein expression levels for native and optimized DNAs in bacterial systems

First author	Optimization algorithm^a^ (source)	Target(s)	Number of constructs	Conclusions
Burgess-Brown [40]	Proprietary (Genscript, Sigma, and MediGene)	Various	30	• 26% of targets show higher expression of soluble protein for optimized over native CDS in *E. coli*
Kudla [27]	CAI	GFP	154	• Fluorescence levels span > 1000-fold across different CDSs • No correlation between fluorescence levels and CAI • Modest relationship between mRNA 2° structure and GFP fluorescence
Welch [28]	PLSR (DNA 2.0)	φ29 DNA polymerase	21	• > 100-fold difference in protein yield observed by differently optimized DNAs
Maertens [41]	CAI (GeneArt)	Various	100	• 24% targets showed ≥ 2× yield for optimized CDS • 20% targets showed lower expression for optimized CDS
Spencer [42]	Undefined	Firefly Luciferase	7	• Optimization increased translation speeds ~ 2× with proportional decrease in functional protein • 2–2.5× yield and solubility increase when recoded for frequent codons in *Drosophila melanogaster*
Trösemeier [43]	CAI (GeneArt) COSEM	ova manA	5 11	• COSEM optimized sequences expressed ≥ 2× the native sequence • “Ramp” inclusion was necessary for significant boost in protein expression
Konczal [44]	CAI (GeneWiz)	KRas4B RalA Rac1	11 11 11	• “Deoptimization” with ≤ 4 rare codons improves solubility ≥ 4× compared to native CDS

*CAI* Codon Adaptation Index, *PLS* partial least squares regression, *COSEM* Codon-Specific Elongation Model

^a^The Kazusa database is reportedly used for codon frequency values by most commercial companies. The COSEM algorithm uses codon frequencies defined by Dong et al. for *E. coli* with a doubling time of 2.5 h^−1^

While hypothesis-driven codon optimization has provided great insight into the complexity of protein expression, we find that the vast majority of published efforts default to various commercial algorithms that offer subsequent synthesis of custom DNAs. We found no published studies that compare commonly used commercial or academic codon optimization algorithms. This deficit led us to ask the question: what are the apparent inequalities of commonly used codon optimization algorithms? Our efforts focused on a single gene, KRas4B, as a case study because the native CDS contains a significant number of rare codons (median codon frequency = 0.34), it is a therapeutically important target implicated in a wide variety of cancers [48], codon usage has been tied to expression levels in bacteria and mammalian cells [44, 49, 50], and there are several reports publishing their CDS with purified yield (enabling correlation of CDS with recombinant product). Moreover, there is an interesting report that investigates the expression levels of KRas4B and the HRas homolog, which natively differ by > 100-fold [49]. These proteins have ~ 85% amino acid identity with only 10% of codons conserved in the native CDS. The researchers showed that conversion of the shared amino acid codons of KRas4B cDNA to those of HRas increased recombinant KRas4B levels ~100× in HEK cells [50]. This conversion also shifted the CAI of KRas4B from 0.69 to 0.87. Recall that a CAI of 1.0 indicates that the codons used for each amino acid are the most abundant.

**Fig. 3:**
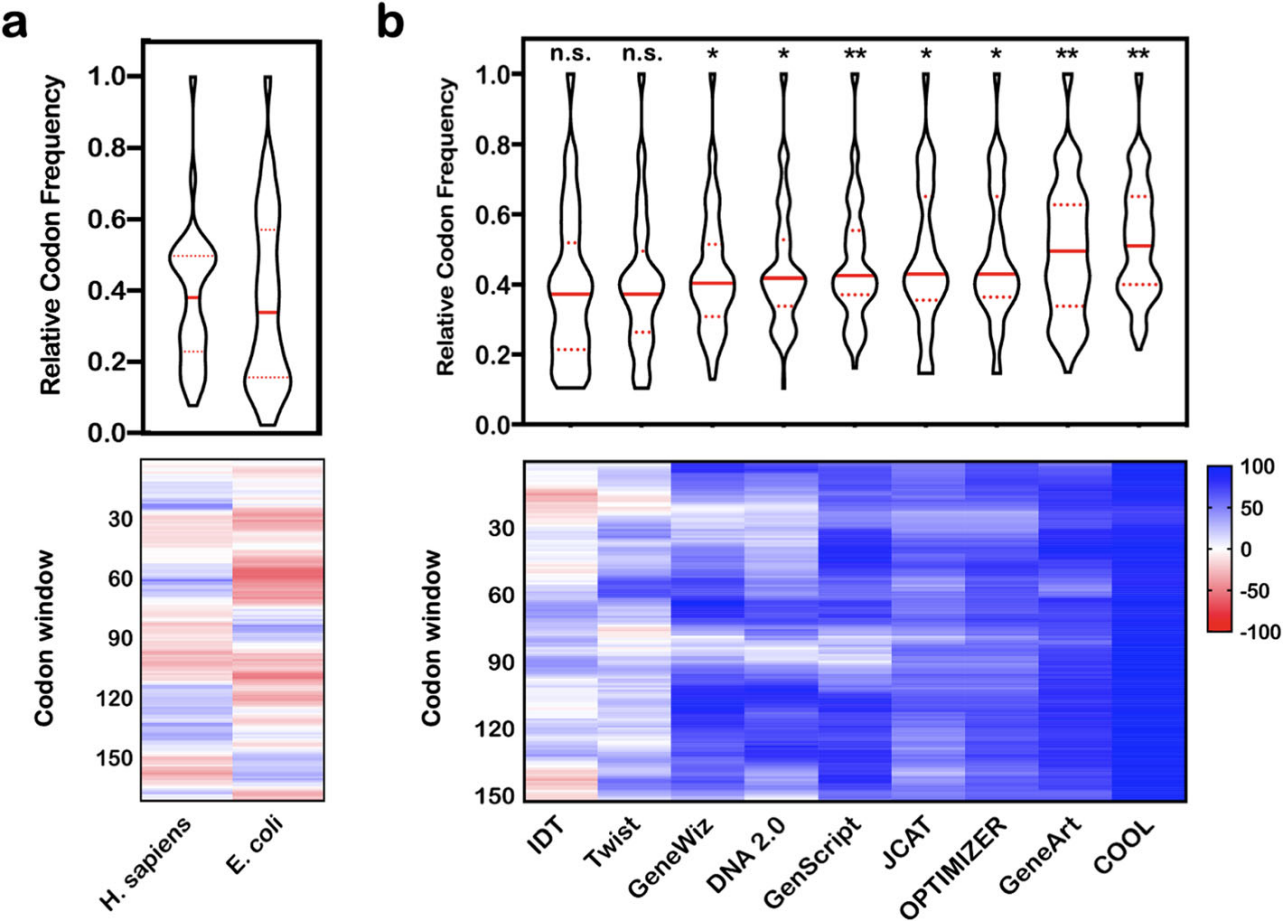
Comparison of the DNA sequences of KRas4B as a function of the relative codon profiles. **a** Violin plot (top) and %MinMax heat map (bottom) of the native CDS (a.a. 1–188) using the codon usage frequencies for *H. sapiens* or *E. coli.* **b** Violin (top) and %MinMax plots (bottom) for KRas4B CDSs (a.a. 1–169) optimized for expression in *E. coli*. Statistical significance was determined with a *Mann-Whitney-Wilcox rank-sum test*: n.s., no significance; *p < 1E−02, **p < 1E−05

The native CDS of KRas4B has been expressed to reasonable levels in BL21 strains of *E. coli* (Table 3). No yields were reported for the aforementioned study in HEK cells [50], but we note the native KRas4B levels were presumably very low (below milligram levels per liter of biomass) because they were detected by Western blotting. It is well known that the ratios for synonymous codons differ greatly between organisms, and we investigate these changes in two ways (Fig. 3): violin plots to visualize the distribution of tabulated RCSU values for all codons used within each CDS and a heat map that represents localized clusters of rare codons using the %MinMax algorithm [34]. These analyses are not commonly employed in protein science and are worth discussing. The violin plots presented here illustrate the relative distribution of RCSU values within a CDS and break the dataset into four sections (each 25% of the total dataset) that are separated by two quartiles (dotted lines) and the median (solid line). The shape of the violin plot represents a normalized distribution of the data, where the widest region signifies the greatest number of data points and the thinnest denotes a minimally populated area of the dataset. In the case of codon optimization, the median/quartiles offer quantitative metrics of changes in the RCSU frequencies while shape changes offer qualitative indicators of shifts within the dataset. The %MinMax analysis uses a sliding window to identify regions within the CDS that are enriched with either frequently (blue) or rarely (red) used codons for a particular organism. Numerical scores range between + 100 and − 100 to represent regions that contain the most frequently or least commonly used codons per amino acid, respectively. A value of zero, illustrated as a white region, indicates an even distribution of codon frequencies. The underlying %MinMax patterns for codon usage clusters are believed to impact the folding rates of the nascent chain as it is translated by the ribosome [53, 54].

**Table 3:** Reported solubility and yield of KRas4B (1–169) from expression in *E. coli*

CDS	N-terminal tag^**a**^	Solubility^**b**^ (%)	Yield (mg/L)	Reference
Native	His_6_	N.R.^e^	11	[51]
	His_8_-28mer-His_8_	41	5	[44]
GeneArt (*n* = 5)	His_6_	38	23 ± 4	This work
DNA2.0	His_6_	N.R.^e^	15	[52]
GeneWiz (opt)^c^	His_8_-28mer-His_8_	24 ± 3	15 ± 2	[44]
GeneWiz (KRas_RARE_)^d^	His_8_-28mer-His_8_	52 ± 22	27 ± 11	[44]

^a^All N-terminal tags were reported to be followed by a TEV protease cleavage site except the GeneArt sequence that contains a dual Thrombin-TEV cleavage site

^b^Solubility measurements represent the normalized ratio of overexpressed protein for the total protein and soluble extract lanes observed in SDS-PAGE analysis

^c^Average and standard deviation for reported values with 0.1–1.0 mM IPTG

^d^KRas_RARE_ represents the average and standard deviation for reported values of the GeneWiz optimized sequence w/ either a single or multiple reintroduced rare codons (I46, I84, R164, I84/R164, and I46/I84/R123/R164)

^e^*N.R.* not reported

Figure 3a compares the codon profiles of the native KRas4B CDS in *Homo sapiens* or *E. coli*. The violin plot shows a nominal change in the median codon frequency (∆ ~ 0.05), but a considerable outward redistribution of the quartiles away from the median when transitioning to a bacterial expression host. Note that the widest portion of the profile also shifts from the 25% quartile to the 75% quartile, indicating a significant rearrangement of the codon profile across expression hosts. The non-normal distribution of these violin plots necessitates that we use a non-parametric method to analyze the statistical significance of this profile shift. We used the Mann-Whitney-Wilcox rank-sum test to assess the similarity of these native CDSs with a *p* value of 0.327. This value indicates that there is little statistical significance between expression hosts despite a striking rearrangement of the %MinMax profile (lower panel) that shows an increased abundance of rare codon clusters indicated by more red regions in the *E. coli* profile. This abundance of rare codon clusters is represented in the violin plot by an increase in codons at or below a frequency of 0.2, although we note that there is no accepted value for defining a rare codon frequency.

Nine optimization algorithms were used to modify the native KRas4B CDS for expression in *E. coli* (Fig. 3b). Qualitative inspection of these violin plots reveals that the widest portion of most datasets lie at or near the median, which is different than the relatively bimodal shape of the native CDS in *H. sapiens*. Furthermore, none of the bacterially-optimized sequences replicate the relative codon or %MinMax profile of the native gene in *H. sapiens*, indicating low harmonization of any optimized CDS with the native DNA profile. Instead, we observe a steady increase in the median codon frequency from 0.34 (native) up to 0.51 (COOL). Relative to the codon frequency of the native DNA, the Mann-Whitney-Wilcox rank-sum analysis of the optimized CDSs shows that two are non-significantly (n.s.) optimized, four exhibit a *p* value between 1.4E−03 and 7.7E−04, and three are highly significant with a *p* < 1E−05 (GenScript, GeneArt, COOL). The statistical significance of the GenScript, GeneArt, and COOL DNAs can be largely attributed to the ranked upward shift of the median codon frequency that results from use of a more even distribution of frequently-to-rarely used codon than other DNAs. Clusters of rare codons are thought to directly influence expression levels or patterns (e.g., translation rates) of the native CDS and consequently may result in misfolded, insoluble, or poorly expressed protein [54]. Visualization of these rare clusters, using the %MinMax algorithm, shows a diminishing trend of rare codon clusters (red bands) to more evenly distributed populations (white bands) and eventually at least one case (COOL) of highly diminished rare codons. The only algorithms that retained rare clusters were the two CDSs (IDT, Twist) that showed no statistical significance for relative codon frequency described above. We conclude that optimization algorithms generally reduce the number and arrangement of rare codons.

Our research group has historically worked with a KRas4B gene synthesized by GeneArt (Life Technologies), which is among the most deviated profiles from the native sequence (Fig. 3). A literature search identified three references that report yields for KRas4B using either the native or an optimized sequence in BL21(DE3) *E. coli*. The general observation is that our yields from the GeneArt DNA are up to 5-fold greater than the native CDS and show a modest gain (1.5×) over published yields using optimized CDSs from GeneWiz and DNA2.0 (Table 3). Konczal et al. report that, despite a 3-fold increase in yield (Table 3), their codon-optimized DNA (GeneWiz) produced lower levels of soluble protein (27%) than the native CDS (41%) [44]. Manipulation of the T7 promoter and alternate start codons showed minimal gains in the overall solubility and often reduced the soluble yields to levels consistent with the native CDS. Significant gains of soluble product were achieved via reintroduction of up to four codons with their respective “rare” (least abundant) codon (KRas_RARE_). Yield gains of the KRas_RARE_ CDSs were an average of sixfold over the native CDS and within error of the product from our GeneArt sequence. Expression of the GeneWiz DNA in Rosetta2 *E. coli*, which overexpress rare codons using the pRARE vector, returned the increased yields from the KRas_RARE_ CDS to levels consistent with the optimized DNA. Konczal et al. therefore concluded that the rare codons were slowing translational kinetics to produce more correctly folded (soluble) KRas4B.

**Fig. 4:**
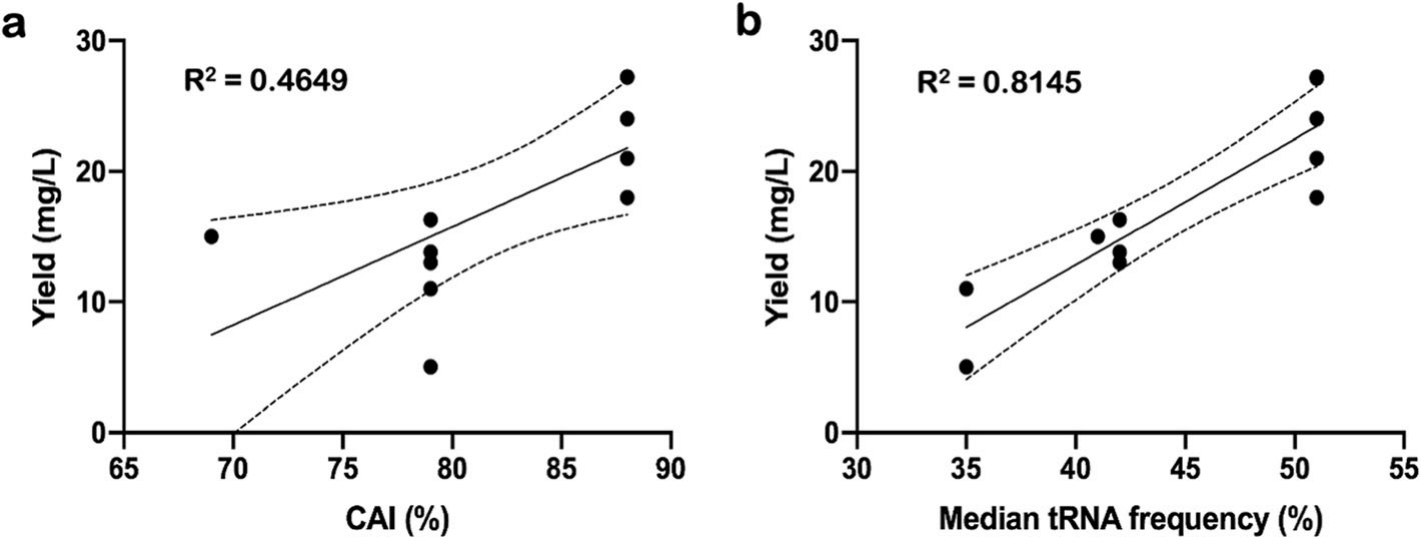
Relationship between soluble proteins yields of KRas4B (a.a. 1–169) with calculated values for **a** CAI or **b** median codon frequency of the CDS. The trendline (solid) is shown with limits for 95% confidence intervals (dotted)

We calculated several commonly used CDS metrics that are used by various optimization algorithms to better understand the relationship between the soluble KRas4B protein yields and their sequences (Table 4). Note that these parameters represent some, but not all, variables considered for optimizing DNAs, and most commercial vendors do not fully disclose their methods. Hence, %GC and %GC3 are factors commonly employed by many algorithms; however, neither value showed a clear correlation of KRas4B CDSs with protein yield (data not shown). CAI also showed poor correlation with protein yield values from Table 3 (Fig. 4a), and remains consistent with previous reports in other protein systems (Table 2). Reasonably good correlations were observed between the protein yield and the median codon frequency of the KRas gene (Fig. 4b), although we are cautious to not overemphasize this relationship as it could vary by protein target. Nevertheless, these data agree with previous reports (Table 2) that indicate CAI may be a poor predictor of protein expression levels [27, 28]. Other metrics, such as median codon frequency, warrant further investigation.

**Table 4:** Variables that are considered for optimized DNA sequences applied to the KRas4B CDS: Median codon frequency, median value for the violin plots shown in Fig. 3; mean frequency, average frequency for the violin plots shown in Fig. 3; CAI, Codon Adaptation Index; %GC, average percent GC content in a DNA sequence; %GC3, average percent GC content in the third (wobble) codon position; %Min window, percent of the DNA sequence that exists with a minimum below zero in the MinMax profile

Vendor algorithm	Median frequency	Mean frequency	CAI	%GC	%GC3	%Min window
Native DNA	0.339	0.370	0.61	37.7	30.2	65
IDT	0.373	0.391	0.68	45.1	50.0	27
Twist	0.373	0.399	0.70	51.5	67.0	17
DNA 2.0^a^	0.404	0.431	0.69	51.8	63.0	0
GeneWiz	0.418	0.446	0.79	54.0	73.6	7
Genscript	0.425	0.458	0.82	54.0	73.6	6
JCAT	0.429	0.470	0.84	46.4	50.0	0
OPTIMIZER	0.429	0.479	0.87	48.0	54.9	0
GeneArt	0.495	0.496	0.88	42.5	37.9	0
COOL	0.509	0.529	0.97	48.1	55.5	0

^a^Reference [52]

### Variable output generated from some algorithms

Our efforts to compare these algorithms led us to test the reproducibility of each server. In principle, we expected that each algorithm should generate a single optimized sequence from multiple inputs of the same native sequence. While most algorithms did so, several servers produced a different output over multiple submissions. Specifically, we submitted the native CDS ten times for three different proteins of increasing length, structural complexity, and various functions: KRas4B (567 bp), Beclin 1 (1350 bp), and PDE3A (3426 bp).

**Fig. 5:**
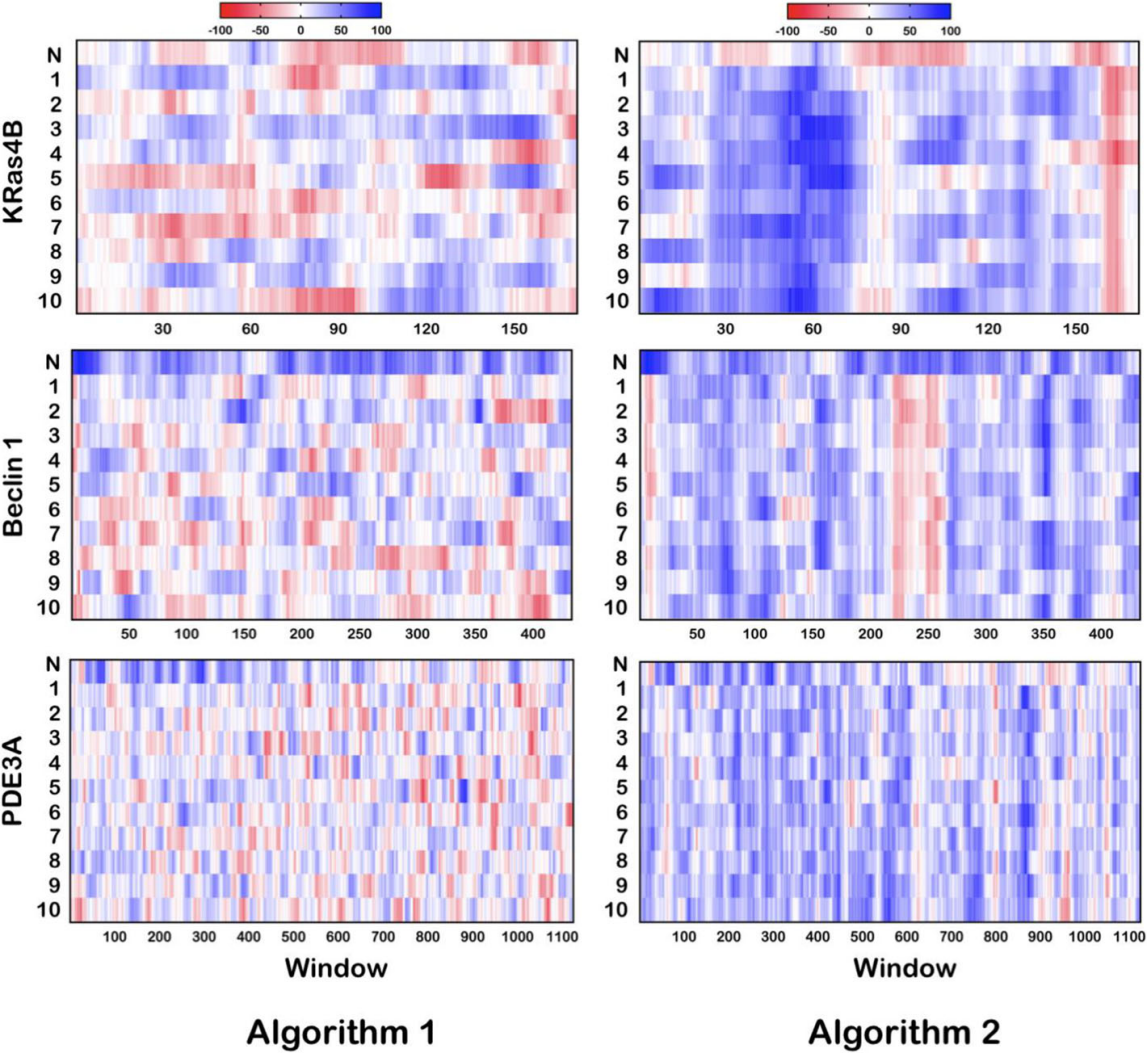
Heat map profiles of proteins optimized by the algorithm from either Algorithm 1 (left) or 2 (right). The codon usage profile of the native sequence (N) was determined with frequencies from *H. sapiens* and the 10 replicates (numbered 1 through 10) were done with frequencies for *E. coli*

**Fig. 6:**
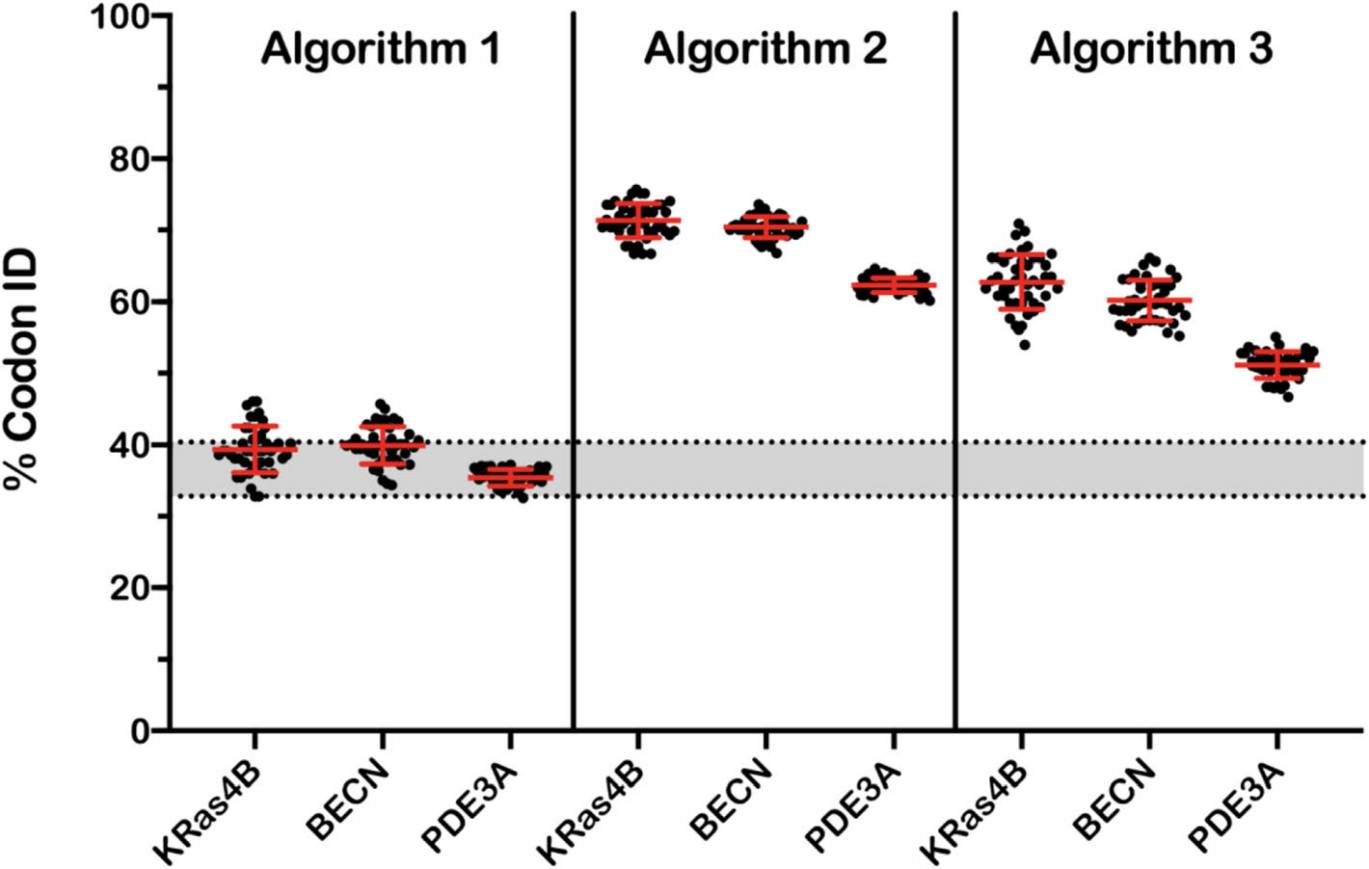
Percentage of codon identity for pairwise alignment of ten DNA sequences optimized by resubmission of the native DNA to a particular optimization algorithm. DNA sequences were from 0.5–3.3 kb. The gray region represents the limits for a random reverse translation for the three different protein sequences (*n* = 100 for each data set)

Three algorithms, in particular, produced strikingly variable output sequences that were best visualized using the %MinMax tool. Results from two algorithms are presented in Fig. 5. Recall that the %MinMax tool represents the linear CDS as a function of codon frequency to identify enriched clusters of rare or frequently used codons. At first glance, it is easy to see there is little consistency in the trends for the optimized profiles of Algorithm 1 and low reproducibility of those for Algorithm 2. We next quantified the pairwise diversity of the 10 optimized sequences of each protein for each algorithm to test how often each algorithm selected a particular codon at a specific position (Fig. 6). A value of 75%, for example, means that 75% of the algorithm-selected codons were reproducibly chosen between optimized sequences. The lower limit of this analysis (33–40%; gray box) represents the range identified for random reverse translation, whereby a set of control sequences were generated by randomly selecting codons for a protein sequence without bias from selection criteria.

Algorithm 1 has an average pairwise identity (35–39%) that is consistent with random reverse translation and raises questions about the validity of the routine. Algorithms 2 and 3 exhibit a higher reproducibility for pairwise alignments (51–70%) and conserves some general codon features throughout the optimized sequences; however, none appear to harmonize well against the native CDS. Such variability raises concerns about how these algorithms enhance DNA sequences and underscores the ambiguous definition of an optimized CDS.

## Discussion

Codon-optimized synthetic DNA sequences have become a normalized resource in recombinant protein work, moving away from efforts with native cDNA. The general hypothesis is that a CDS tailored for a particular expression system will yield higher levels of recombinant protein than a non-optimized cDNA. While there exist many examples of improved expression levels for proteins of various sizes and functions, manipulation of the CDS is also reported to have a negative effect on protein yields and solubility (Table 2). This observation led us to investigate and develop a method for comparing the relative inconsistencies between commonly used, publicly available codon optimization algorithms.

The first step involved recognizing that many commonly used optimization algorithms enhance DNAs around the CAI metric, which appears to have shifted from using a handful of highly expressing genes in *E. coli* to being replaced by organism-specific codon frequency tables. We identified that the most widely used codon frequency source comes from the outdated Kazusa database [9] and showed that newer databases with more comprehensive genetic libraries present different codon biases (Table 1 and Fig. 2). To his end, we chose the HIVE-CUT database to tabulate codon frequencies of DNAs manipulated by nine different codon optimization algorithms using the well-characterized KRas4B oncogene as a case study. Visualization of these distributions through the use of violin plots or the %MinMax routine shows the relative enrichment or depletion of codon populations throughout the gene (Fig. 3). Violin plots specifically enable researchers with a new metric for quickly assessing codon usage frequencies to identify the relative bias across optimization algorithms. While the %MinMax tool was originally developed for identification of rare codon clusters [54], we find that pairing these plots with violin plots of codon frequency furthers evaluation of DNA optimization by mapping regions that are commonly enriched or depleted throughout the linear gene. Note that none of the optimized CDSs (Fig. 3b) replicate the distribution of the native DNA in *H. sapiens* (Fig. 3a). Instead, we observe that only two algorithms, GeneArt and COOL, generate DNAs with a significant increase in codons having a higher usage frequency (Fig. 3b, upper panel) and depletion of rare codon clusters throughout the gene (Fig. 3b, lower panel). Comparison of three optimized DNAs to the native CDS demonstrates that the upward trend of the median codon frequency correlates better with purified yields of bacterially expressed KRas4B than the commonly used CAI metric (Fig. 4).

### Publishing optimized sequences as a community effort

Recall that there is currently little-to-no consensus for what features define an optimized DNA sequence. We therefore recommend that researchers publish their optimized DNA sequences and report functional yields for three simple reasons. First, there is sufficient evidence that 20–25% of optimized DNAs result in increased yields relative to the native CDS and at least one report of diminished yields from ~ 20% of optimizations (Table 2). Researchers should therefore consider comparing expression levels of the native CDS with an optimized sequence. Having the native sequence in hand can also save time and resources by quickly transitioning into other expression systems, like insect or mammalian cell lines, without needing reoptimization for usage in these systems. Second, while most studies report the commercial vendor used to synthesize their DNAs, we should not expect that the optimization algorithms used in a publication will generate the same CDS upon resubmission. We demonstrated that at least three algorithms produce different outputs from the same input DNA upon replicate submissions (Figs. 5 and 6). This inconsistency is of particular concern when paired with the roughly equivalent chance of improved or diminished yields discussed above. Lastly, publication of DNA sequences will only improve the reproducibility of protein production, which is a well-documented problem in many scientific fields [55]. Publishing these sequences is rare in an age where Supplemental Information is supplied with most scientific reports. Moreover, plasmid repositories (e.g., Addgene) and databases (e.g., DNASU, SGDB) have been developed for this purpose [19, 20].

### Practical considerations

We have presented a new method for comparing different codon-optimized CDSs, as each can result in widely varied yields in the recombinant target protein. We recommend the following list of steps and considerations when working with codon-optimized DNAs. First, collect several organism-specific, codon-optimized DNAs from several commercial and academic algorithms. We anticipate that most publicly available algorithms will evolve over time and should be evaluated for every new target. Second, identify an updated source for quantifying codon usage frequencies. The Kazusa database is the most widely used source of organism-specific codon usage tables but was last updated in 2007 [9]. We used the HIVE-CUT database for the work presented here [10], but realize that there may be better sources for other applications. Third, assess the relative codon frequency distribution of all optimized DNAs using violin plots to identify shifts in the relative codon population and median frequency, and the %MinMax tool to detect any enriched or depleted regions of codon clusters. Fourth, select an optimized DNA that aligns with a testable hypothesis. In the KRas4B example presented here, we found that the highest yields of soluble protein were obtained with DNA optimized for a statistically significant increase in relative codon frequency (*p* < 1E−05) and depletion of rare codon clusters. Lastly, and most importantly, disseminate the optimized DNAs used in any published work related to the construct, either in the supporting materials that accompany a publication or plasmid repositories. This effort, at a minimum, will aid reproducibility for preparing recombinant targets as even small changes in a CDS can have large impacts on yields [44]. Replicating optimized sequences from a published source may also be difficult because algorithms likely change over time or result in variable sequences (Figs. 5 and 6). The sequences used in this work are presented in the supporting information (Additional file 1, Table 4).

## Conclusions

We constructed this report to enable researchers with a set of tools and recommendations for evaluating the numerous options for codon optimizing DNAs. The specific combination of violin plots and the %MinMax tool [54] provide a straightforward visual alternative to the CAI metric that is commonly employed by many popular algorithms. These tools are especially useful for quickly identifying which algorithms may enrich or deplete rare codons (Fig. 3) and, upon replicate submissions, which algorithms generate variable output sequences (Figs. 5 and 6). Better transparency of the successful CDSs, through publication of DNA sequences as supplementary materials or by depositing them in repositories, will improve reproducibility across scientific fields. We also recommend that the more comprehensive HIVE-CUT database replace the Kazusa database for organism-specific codon usage tables (Fig. 2 and Table 1). Genomic data has significantly improved since the last update of the Kazusa database in 2007 and we believe that the field should transition to newer, more representative codon usage datasets for the analyses presented in this work and codon optimization in general.

## Methods

### Sequences

Native CDSs for KRas4B (Accession: AF493917), Beclin 1 (Accession: NM_003766), and PDE3A (Accession: NM_000921) were collected from the NCBI database. Commercial or academic online servers were used to generate optimized CDSs for *E. coli*. All native and optimized CDSs are provided as Supplementary Data (Additional file 1, Tables 3 and 4).

### Calculations

RCSU values were tabulated for six databases according to Sharp and Li [8]. CAI calculations were done using CAICal [56]. Codon frequencies for RCSU and CAI calculations used tables for *E. coli* from the HIVE-CUT database [10]. The %GC and %GC3 content of CDSs was calculated using E-CAI server [57]. The reproducibility of each server was quantified by first submitting the native CDS as unique entries up to ten times, collecting the optimized sequence from each entry, and calculating pairwise alignments between optimized sequences to determine the “% Codon Identity.” A set of random reverse translation sequences, which assumes no codon bias, was created from amino acid sequences to determine the lower limit of the “% Codon Identity” analysis. The %MinMax server was used to assess how native and optimized CDSs distribute rare or commonly used codons throughout the gene based on the HIVE-CUT codon usage table for *E. coli* [54]. The generation and statistical analysis of violin plots was done using Prism 8 (GraphPad Software, Inc.).

### Expression and purification of the GeneArt optimized KRas4B CDS

The native CDS of KRas4B was codon-optimized for expression in *E. coli* (GeneArt, Life Technologies) and cloned into a pET21b vector with a N-terminal His_6_-tag for purification. The expression vector was transformed into One Shot BL21(DE3) *E. coli* (ThermoFisher). Large scale expression of KRas4B was performed by inoculating 1 L of TB with a starter culture (1:1000) and ampicillin (100 μg/mL), growing the culture at 37 °C with orbital shaking at 180 rpm to mid-log phase (A_600_ ~ 0.8), cooling cultures to 18 °C for 30 min prior to inducing overexpression with 0.5 mM IPTG, and allowing cultures to continue growth at 18 °C for 16–18 h. Cultures were harvested and stored at − 80 °C until purification.

Purification of KRas4B began by resuspending cells in lysis buffer [50 mM Tris-HCl (pH 8.0), 500 mM NaCl, 20 mM imidazole, 10% glycerol, 0.5 mM TCEP, 1× lysonase, 1x Roche Protease Inhibitor (EDTA free)] and sonicating on ice. Lysates were clarified via centrifugation and filtered (0.2 μm) before loading onto a Ni^2+^-charged HisTrap column (GE Life Sciences). The column was washed with wash buffer [50 mM Tris-HCl (pH 8.0), 500 mM NaCl, 10% glycerol, 5 mM MgCl_2_, 0.5 mM TCEP] containing 20 mM imidazole for 5 column volumes before eluting with an imidazole gradient. Fractions containing the eluted KRas4B protein were pooled and dialyzed overnight in wash buffer (1:1000) in the presence of TEV protease. The digested product was separated from TEV protease and uncleaved KRas4B by passing the dialyzed sample over a Ni^2+^-charged HisTrap column and collected as the flow through fraction. The cleaved KRas4B product was concentrated and passed over a Superdex 75 26/600 GL column (GE Life Sciences) using an isocratic elution in SEC buffer [25 mM HEPES (pH 7.4), 150 mM NaCl, 5 mM MgCl_2_, 10% glycerol, 0.5 mM TCEP]. SDS-PAGE was used to assess the relative purity of the eluted fractions. The mass of KRas4B (calculated mass, 19,303 Da; observed mass, 19,302 Da) was confirmed by LC/MS. KRas4B containing fractions were pooled, concentrated, quantified using a Bradford assay, and stored at − 80 °C.

## Supplementary Information

**Additional file 1 **Spreadsheet containing DNA sequences and tabulated data presented in this work. **Table 1.** Raw and normalized counts for human recombinant proteins from different expression systems that were collected from the RCSB PDB on July 23, 2019. **Table 2.** RCSU data for Fig. 2 heat map describing codon frequencies in *E. coli*. **Table 3.** KRas4B Sequences from various algorithms; and Data for Fig. 3 violin plots and %MinMax heatmaps for native and vendor algorithm optimized CDSs. **Table 4.** DNA outputs from optimization algorithms collected while testing reproducibility for three proteins.

## Data Availability

All data generated and used in this work, including DNA sequences and codon tables used, are available in Additional file 1: Supplementary Information. Python scripts used for the analysis of sequence data presented in this work are available at Github [58].

## Abbreviations

CAI: Codon Adaptation Index
CDS: Coding DNA sequence
COSEM: Codon-Specific Elongation Model
DNASU: DNA repository at Arizona State University
HIVE-CUT: High-Performance Integrated Virtual Environment Codon Usage Tables
PDB: Protein Data Bank
PLSR: Partial least square regression
RCSB: Research Collaboratory for Structural Bioinformatics
RCSU: Relative synonymous codon usage
SGDB: Synthetic gene database

## References

[1] Parret A, Besir H, Meijers R (2016). Critical reflections on synthetic gene design for recombinant protein expression. Curr Opin Struct Biol.

[2] Rosano G, Morales ES, Ceccarelli EA. New tools for recombinant protein production in *Escherichia coli*: a 5-year update. Protein Sci. 2019;28:1412–22. 10.1002/pro.3668 PMC6635841 31219641

[3] Sivashanmugam A, Murray V, Cui C, Zhang Y, Wang J, Li Q. Practical protocols for production of very high yields of recombinant proteins using *Escherichia coli*. Protein Sci. 2009;18:936–48. 10.1002/pro.102 PMC2771296 19384993

[4] Studier FW (2005). Protein production by auto-induction in high density shaking cultures. Protein Expr Purif.

[5] Paraskevopoulou V, Falcone FH. Polyionic tags as enhancers of protein solubility in recombinant protein expression. Microorganisms. 2018;6:e20047. 10.3390/microorganisms6020047 PMC6027335 29882886

[6] Maina CV, Riggs PD, Grandea AG, Slatko BE, Moran LS, Tagliamonte JA, McReynolds LA, di Guan C. An *Escherichia coli* vector to express and purify foreign proteins by fusion to and separation from maltose-binding protein. Gene. 1988;74:365–73. 10.1016/0378-1119(88)90170-9 3073105

[7] Novoa EM, de Pouplana LR. Speeding with control: codon usage, tRNAs, and ribosomes. Trends Genet. 2012;28:574–81. 10.1016/j.tig.2012.07.006 22921354

[8] Sharp PM, Li W-H. Codon usage in regulatory genes in Escherichia coli does not reflect selection for ‘rare’ codons. Nucleic Acids Res. 1986;14:7737–49. 10.1093/nar/14.19.7737 PMC311793 3534792

[9] Nakamura Y, Gojobori T, Ikemura T. Codon usage tabulated from international DNA sequence databases: status for the year 2000. Nucleic Acids Res. 2000;28:292. 10.1093/nar/28.1.292 PMC102460 10592250

[10] Athey J, Alexaki A, Osipova E, Rostovtsev A, Santana-Quintero LV, Katneni U, Simonyan V, Kimchi-Sarfaty C (2017). A new and updated resource for codon usage tables. BMC Bioinformatics.

[11] Quax TEF, Claassens NJ, Soll D, can der Oost J (2015). Codon Bias as a means to fine-tune gene expression. Mol Cell.

[12] Chan PP, Lowe TM (2009). GtRNAdb: a database of transfer RNA genes detected in genomic sequence. Nucleic Acids Res.

[13] Dong H, Nilsson L, Kurland CG. Co-variation of tRNA abundance and codon usage in *Escherichia coli* at different growth rates. J Mol Biol. 1996;260:649–63. 10.1006/jmbi.1996.0428 8709146

[14] Koblan LW, Doman JL, Wilson C, Levy JM, Tay T, Newby GA, Maianti JP, Raguram A, Liu DR. Improving cytidine and adenine base editors by expression optimization and ancestral reconstruction. Nat Biotechnol. 2018;36:843–6. 10.1038/nbt.4172 PMC6126947 29813047

[15] Sauna ZE, Kimchi-Sarfaty C (2011). Understanding the contribution of synonymous mutations to human disease. Nat Rev.

[16] Mauro VP, Chappell SA. A critical analysis of codon optimization in human therapeutics. Trends Mol Med. 2014;20:604–13. 10.1016/j.molmed.2014.09.003 PMC4253638 25263172

[17] Wu G, Dress L, Freeland SJ (2007). Optimal encoding rules for synthetic genes: the need for a community effort. Mol Syst Biol.

[18] Mauro VP, Chappell SA. Considerations in the use of codon optimization for recombinant protein expression. In: Hacker DL, editor. Recombinant Protein Expression in Mammalian Cells: Methods and Protocols, vol. 1850. New York: Springer Nature; 2018. p. 275–88. 10.1007/978-1-4939-8730-6_18 30242693

[19] Wu G, Zheng Y, Qureshi I, Zin HT, Beck T, BUlka B, Freeland SJ. SGDB: a database of synthetic genes re-designed for optimizing protein over-expression. Nucleic Acids Res. 2007;35:D76–9. 10.1093/nar/gkl648 PMC1781117 17062619

[20] Seiler CY, Park JG, Sharma A, Hunter P, Surapaneni P, Sedillo C, Field J, Algar R, Price A, Steel J (2014). DNASU plasmid and PSI:biology-materials repositories: resources to accelerate biological research. Nucleic Acids Res.

[21] Gibson DG, Glass JI, Lartigue C, Noskov VN, Chuang RY, Algire MA, Benders GA, Montague MG, Ma L, Moodie MM, et al. Creation of a bacterial cell controlled by a chemically synthesized genome. Science. 2010;329:52–6. 10.1126/science.1190719 20488990

[22] Gibson DG, Benders GA, Andrews-Pfannkoch C, Denisova EA, Baden-Tillson H, Zaveri J, Stockwell TB, Brownley A, Thomas DW, Algire MA (2008). Complete chemical synthesis, assembly, and cloning of a mycoplasma genitalium genome. Science.

[23] Sharp PM, Li W-H. The codon adaptation index - a measure of directional synonymous codon usage bias, and its potential applications. Nucleic Acids Res. 1987;15(3):1281–95. 10.1093/nar/15.3.1281 PMC340524 3547335

[24] dos Reis M, Wenisch L, Savva R. Unexpected correlations between gene expression and codon usage bias from microarray data for the whole Escherichia coli K-12 genome. Nucleic Acids Res. 2003;31:6976–85. 10.1093/nar/gkg897 PMC290265 14627830

[25] Wright F. The ‘effective number of codons’ used in a gene. Gene. 1990;87:23–9. 10.1016/0378-1119(90)90491-9 2110097

[26] Plotkin JB, Kudla G. Synonymous but not the same: the causes and consequences of codon bias. Nat Rev Genet. 2011;12:32–43. 10.1038/nrg2899 PMC3074964 21102527

[27] Kudla G, Murray AW, Tollervey D, Plotkin JB. Coding-sequence determinants of gene expression in *Escherichia coli*. Science. 2009;324:255–8. 10.1126/science.1170160 PMC3902468 19359587

[28] Welch M, Govindarajan S, Ness JE, Villalobos A, Gurney A, Minshull J, Gustafsson C. Design parameters to control synthetic gene expression in *Escherichia coli*. PLoS One. 2009;4:e7002. 10.1371/journal.pone.0007002 PMC2736378 19759823

[29] Alexaki A, Kames J, Holcomb DD, Athey J, Santana-Quintero LV, Lam PV, Hamasaki-Karagiri N, Osipova E, Simonyan E, Bar H, et al. Codon and codon-pair usage tables (CoCoPUTs): facilitating genetic variation analyses and recombinant Gene Design. J Mol Biol. 2019;431:2434–41. 10.1016/j.jmb.2019.04.021 31029701

[30] Goodman DB, Church GM, Kosuri S (2013). Causes and effects of N-terminal codon bias in bacterial genes. Science.

[31] Mathews DH, Disney MD, Childs JL, Schroeder SJ, Zuker M, Turner DH. Incorporating chemical modification constraints into a dynamic programming algorithm for prediction of RNA secondary structure. Proc Natl Acad Sci U S A. 2004;101:7287–92. 10.1073/pnas.0401799101 PMC409911 15123812

[32] Li GW, Oh E, Weissman JS. The anti-Shine-Dalgarno sequence drives translational pausing and codon choice in bacteria. Nature. 2012;484:538–41. 10.1038/nature10965 PMC3338875 22456704

[33] Komar AA. The Yin and Yang of codon usage. Hum Mol Genet. 2016;25:R77–85. 10.1093/hmg/ddw207 PMC6372012 27354349

[34] Clarke TF IV, Clark PL. Rare codons cluster. PLoS One. 2008;3:e3412. 10.1371/journal.pone.0003412 PMC2565806 18923675

[35] Gustafsson C, Govindarajan S, Minshull J. Codon bias and heterologous protein expression. Trends Biotechnol. 2004;22:346–53. 10.1016/j.tibtech.2004.04.006 15245907

[36] Rosano GL, Ceccarelli EA. Rare codon content affects the solubility of recombinant proteins in a codon bias-adjusted *Escherichia coli* strain. Microb Cell Factories. 2009;8:41–50. 10.1186/1475-2859-8-41 PMC2723077 19630980

[37] Fath S, Bauer AP, Liss M, Spriestersbach A, Maertens B, Hahn P, Ludwig C, Scha ¨fer F, Graf M, Wagner R. Multiparameter RNA and codon optimization: a standardized tool to assess and enhance autologous mammalian gene expression. PLoS One. 2011;6:e17596. 10.1371/journal.pone.0017596 PMC3048298 21408612

[38] Raab D, Graf M, Notka F, Schodl T, Wagner R (2010). The GeneOptimizer algorithm: using a sliding window approach to cope with the vast sequence space in multiparameter DNA sequence optimization. Syst Synth Biol.

[39] Villalobos A, Ness JE, Gustafsson C, Minshull J, Govindarajan S. Gene designer: a synthetic biology tool for constructing artificial DNA segments. BMC Bioinformatics. 2006;6:285. 10.1186/1471-2105-7-285 PMC1523223 16756672

[40] Burgess-Brown NA, Sharma S, Sobott F, Loenarz C, Oppermann U, Gileadi O. Codon optimization can improve expression of human genes in *Escherichia coli*: a multi-gene study. Protein Expr Purif. 2008;59:94–102. 10.1016/j.pep.2008.01.008 18289875

[41] Maertens B, Spriestersbach A, von Groll U, Roth U, Kubicek J, Gerrits M, Graf M, Liss M, Daubert D, Wagner R, et al. Gene optimization mechanisms: a multi-gene study reveals a high success rate of full-length human proteins expressed in *Escherichia coli*. Protein Sci. 2010;19:1312–26. 10.1002/pro.408 PMC2970903 20506237

[42] Spencer PS, Siller E, Anderson JF, Barral JM (2012). Silent substitutions predictably alter translation elongation rates and protein folding efficiencies. J Mol Biol.

[43] Trösemeier J-H, Rudorf S, Loessner H, Hofner B, Reuter A, Schulenborg T, Koch I, Bekeredjian-Ding I, Lipowsky R, Kamp C (2019). Optimizing the dynamics of protein expression. Sci Rep.

[44] Konczal J, Bower J, Gray CH. Re-introducing non-optimal synonymous codons into codon-optimized constructs enhances soluble recovery of recombinant proteins from *Escherichia coli*. PLoS One. 2019;14:e0215892. 10.1371/journal.pone.0215892 PMC6478350 31013332

[45] Chaney JL, Steele A, Carmichael R, Rodriguez A, Specht AT, Ngo K, Li J, Emrich S, Clark PL (2017). Widespread position-specific conservation of synonymous rare codons within coding sequences. PLoS Comput Biol.

[46] Jacobs WM, Shakhnovich EI. Evidence of evolutionary selection for cotranslational folding. Proc Natl Acad Sci USA. 2018;114:11434–9. 10.1073/pnas.1705772114 PMC5664504 29073068

[47] Angov E, Hillier CJ, Kincaid RL, Lyon JA. Heterologous protein expression is enhanced by harmonizing the codon usage frequencies of the target gene with those of the expression host. PLoS One. 2008;3:e2189. 10.1371/journal.pone.0002189 PMC2364656 18478103

[48] Prior IA, Lewis PD, Mattos C. A comprehensive survey of Ras mutations in cancer. Cancer Res. 2012;72:2457–67. 10.1158/0008-5472.CAN-11-2612 PMC3354961 22589270

[49] Lampson BL, Pershing NL, Prinz JA, Lacsina JR, Marzluff WF, Nicchitta CV, MacAlpine DM, Counter CM. Rare codons regulate KRas oncogenesis. Curr Biol. 2013;23:70–5. 10.1016/j.cub.2012.11.031 PMC3567844 23246410

[50] Fu J, Dnag Y, Counter C, Liu Y. Codon usage regulates human KRAS expression at both transcriptional and translational levels. J Biol Chem. 2018;293:17929–40. 10.1074/jbc.RA118.004908 PMC6240855 30275015

[51] Biancucci M, Dolores JS, Wong J, Grimshaw S, Anderson WF, Satchell KJF, Kwon K. New ligation independent cloning vectors for expression of recombinant proteins with a self-cleaving CPD/6xHis-tag. BMC Biotechnol. 2017;17:1–11. 10.1186/s12896-016-0323-4 PMC5216533 28056928

[52] Hunter JC, Manandhar A, Carrasco MA, Gurbani D, Gondi S, Westover KD. Biochemical and structural analysis of common cancer-associated KRAS mutations. Mol Cancer Res. 2015;13:1325–35. 10.1158/1541-7786.MCR-15-0203 26037647

[53] Sander IM, Chaney JL, Clark PL (2014). Expanding Anfinsen’s principle: contributions of synonymous codon selection to rational protein design. J Am Chem Soc.

[54] Rodriguez A, Wright G, Emrich S, Clark PL (2017). %MinMax: a versatile tool for calculating and comparing synonymous codon usage and its impact on protein folding. Protein Sci.

[55] Yaffe MB. Reproducibility in science. Sci Signal. 2015;8:eg5. 10.1126/scisignal.aaa5764 25852185

[56] Puigbò P, Bravo IG, Garcia-Vallve S (2008). CAIcal: a combined set of tools to assess codon usage adaptation. Biol Direct.

[57] Puigbo P, Bravo IG, Garcia-Vallve S (2008). E-CAI: a novel server to estimate an expected value of Codon Adaptation Index (eCAI). BMC Bioinformatics.

[58] Li, JJ: Codon Optimization Analysis Tools [https://github.com/JeffreyJLi/codon_optimization_analysis] Accessed 22 May 2020.

